# PlantMP: a database for moonlighting plant proteins

**DOI:** 10.1093/database/baz050

**Published:** 2019-04-25

**Authors:** Bo Su, Zhuang Qian, Tianshu Li, Yuwei Zhou, Aloysius Wong

**Affiliations:** 1 Department of Computer Science, College of Science and Technology, Wenzhou-Kean University, 88 Daxue Road, Ouhai, Wenzhou, Zhejiang Province, China; 2 Department of Biology, College of Science and Technology, Wenzhou-Kean University, 88 Daxue Road, Ouhai, Wenzhou, Zhejiang Province, China

## Abstract

Moonlighting proteins are single polypeptide chains capable of executing two or more distinct biochemical and/or biological functions. Here, we describe the development of PlantMP, which is a manually curated online-based database of plant proteins that are known to `moonlight’. The database contains searchable UniProt IDs and names, canonical and moonlighting functions, gene ontology numbers, plant species as well as links to the PubMed indexed articles. Proteins homologous to experimentally confirmed moonlighting proteins from the model plant *Arabidopsis thaliana* are provided as a separate list of `likely moonlighters’. Additionally, we also provide a list of predicted Arabidopsis moonlighting proteins reported in the literature. Currently, PlantMP contains 110 plant moonlighting proteins, 10 `likely moonlighters’ and 27 `predicted moonlighters’. Organizing plant moonlighting proteins in one platform enables researchers to conveniently harvest plant-specific raw and processed data such as the molecular functions, biological roles and structural features essential for hypothesis formulation in basic research and for biotechnological innovations.

## Introduction

Moonlighting proteins are single polypeptide chains capable of executing two or more distinct biochemical and/or biological functions. Unlike multi-protein complexes, they are not products of gene fusions, RNA splice variants or proteolytic fragments. They also exclude pleiotropic proteins and members of the same protein family that perform different functions (1). Moonlighting proteins are found in organisms across the tree of life where they assume important secondary functions such as regulating transcription, catalysis and tuning signaling networks (2–8). These alternative functions confer cost- and efficiency-associated benefits by making fewer proteins from a more compact genome and increase control of regulatory and signaling pathways (9). However, moonlighting functions of proteins are often discovered by serendipity, thus the creation of moonlighting protein databases such as MoonProt (10) and MultitaskProtDB (11) to pool together this class of proteins especially given that they are increasingly common and have been implicated in human diseases (9).

The requirement for spatial and temporal micro-regulations of cellular biochemicals such as signaling molecules, ligands and cofactors becomes more significant considering the relatively crowded space of the plant cell, which is occupied by large central vacuoles (12). Many canonical domains in other organisms, including animals and bacteria that exist as stand-alone proteins, have been incorporated into multi-functional complex plant proteins often as smaller functional centers that are embedded within larger primary domains (13). Indeed, there have been well-characterized examples in receptor protein complexes such as BRI1 from *Arabidopsis thaliana* where externally located hormone recognition domain is connected via a transmembrane region to a cytoplasmic canonical kinase domain that also accommodates a smaller moonlighting guanylate cyclase (GC) center (14). The same is also observed in Arabidopsis potassium transporter and channel (KUP5 and GORK) proteins, where moonlighting adenylate cyclase (AC) center and ABA binding site were found to occupy cytosolic sites of these multi-pass membrane proteins (15, 16). This complexity also makes it incredibly difficult to examine precise cellular regulatory functions of individual components and their broader biological roles.

Current databases contain moonlighting proteins from human and other organisms, but plant moonlighters are under-represented. The fundamental differences in domain architecture and cellular organization of plant proteins would require a more focused avenue to aid discovery of similar proteins, data interpretation and annotation of moonlighting proteins in model plants, crops and other economically valuable plants. A comprehensive catalog of plant moonlighting proteins can therefore better serve the plant science community in sequencing, proteomics and structural works, thus the creation of PlantMP.

## Materials and methods

### Selection of proteins for inclusion in PlantMP database

**Figure 1:**
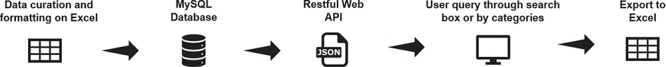
PlantMP database and interface. Information extracted from various databases and articles was manually curated and formatted offline on Excel before importing into MySQL database in the following format: protein name, gene uniprot_id, canonical_function_bp, canonical_function_mf, moonlighting_function_bp, moonlighting_function_mf, go_canonical_bp, go_canonical_mf, go_moonlighting_bp, go_moonlighting_mf, plant_species, pubmed_id, type. `Type’ indicates if the moonlighting function of the protein is predicted, likely or confirmed based on the literature. Database website structure was designed and developed using the front-end and back-end separation, which has the benefit of low-degree coupling. The website front-end will query the database when user invoked either the search function by UniProt ID, protein name or plant species or by selecting one of the four categories provided. At the back-end, data is provided using Restful Web API with JSON format. For example, all moonlighting proteins from *A. thaliana* can be retrieved by querying the URL https://www.plantmp.com/api/species?species=Arabidopsis in the web browser. For website development, an online web template provided by Colorlib (http://www.cssmoban.com/cssthemes/8065.shtml) was adopted and modulated with feature module for database query. Front-end only requires the setting up of the user query input as a parameter to specify API URL and then displayed in appropriate table HTML format. Users have the option to export all or selected results into Excel format.

**Figure 2:**
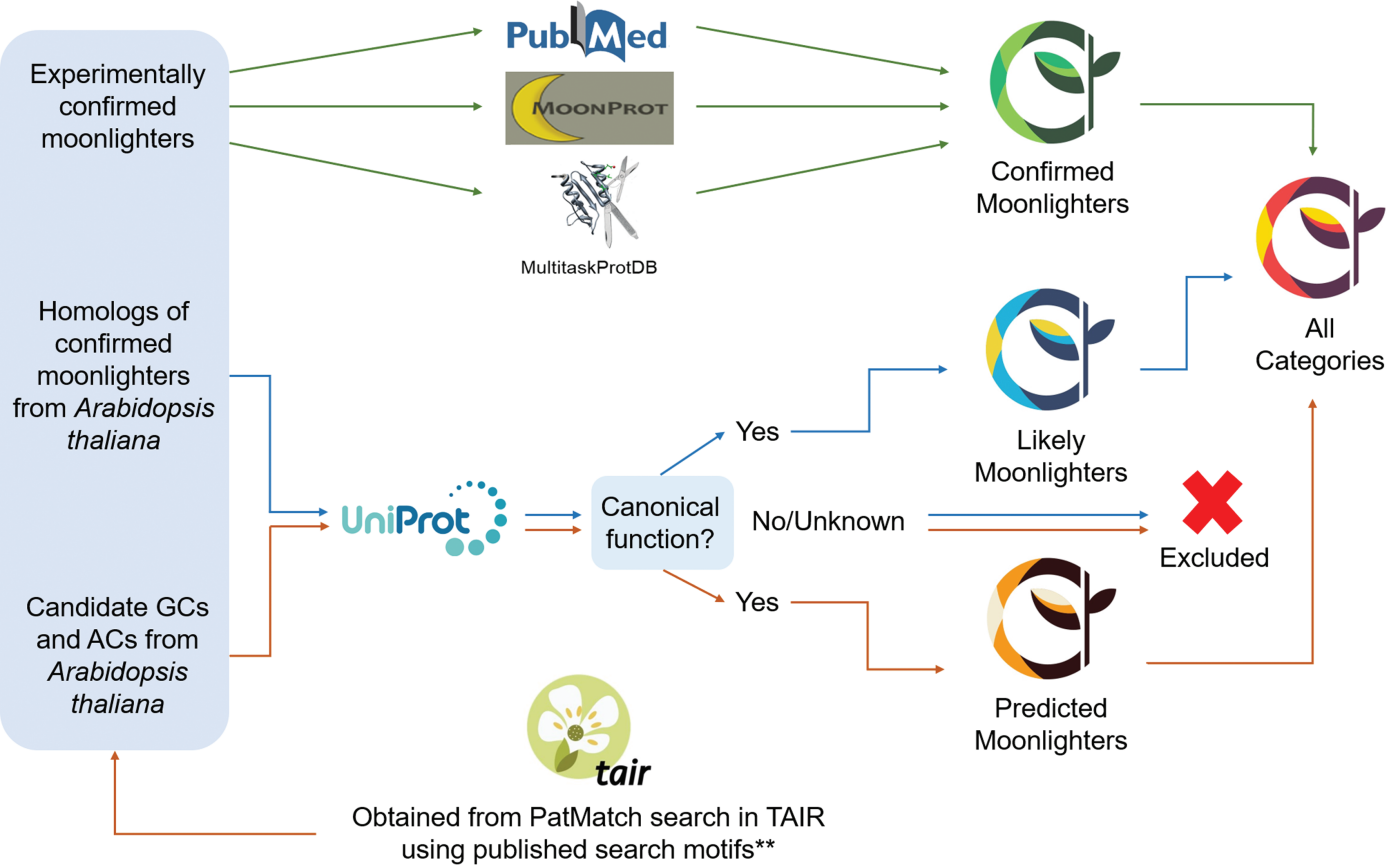
Selection of moonlighting plant proteins for inclusion in PlantMP database. Proteins with experimentally validated moonlighting functions retrieved from PubMed and other databases such as MoonProt (http://moonlightingproteins.org) (10) and MultiTaskProtDB (http://wallace.uab.es/multitaskII) (11) were included in the list of `confirmed moonlighters’ (indicated as green arrows). Homologs of confirmed moonlighters from *A. thaliana* were included in the list of `likely moonlighters’ (indicated as blue arrows), while reported candidate GC and AC (which are typically moonlighting in multi-domain plant proteins) from *A. thaliana* (13) were included in the list of `predicted moonlighters’ (indicated as orange arrows). Both `likely’ and `predicted’ moonlighters were only included if they have known primary functions and harbor domains or motifs that could hint moonlighting functions based on UniProt annotations (17). The candidate GCs and ACs were retrieved from a PatMatch search on TAIR (https://www.arabidopsis.org/cgi-bin/patmatch/nph-patmatch.pl) (28) using search motifs **reported by (13, 20).

Experimentally validated plant moonlighting proteins were collected from PubMed using `plant moonlighting proteins’ and `dual/double function/role plant protein’ as the search terms. Retrieved articles were manually screened and information of plant moonlighting proteins were extracted from the articles for inclusion in the PlantMP database. Homologs of experimentally validated moonlighting proteins from *A. thaliana* were given as a separate list of `likely moonlighters’. Additionally, a list of `predicted moonlighters’ that consist of Arabidopsis proteins, which are not homologs of known moonlighting proteins but have been reported to be candidate GCs or ACs, were included in the database. In both `likely moonlighters’ and `predicted moonlighters’, their canonical functions were first determined on UniProt (17), and if no primary functions are known, they are excluded from the database. Additionally, we also checked proteins in the list of `likely moonlighters’ for the presence of known domains or motifs that could hint moonlighting functions (Figures 1 and 2).

### Protein information included in PlantMP database

Moonlighting plant proteins in the PlantMP database contain searchable UniProt IDs and names, canonical and moonlighting functions, gene ontology (GO) numbers, plant species and links associated to the PubMed articles. Included in the canonical and moonlighting functions are their molecular function (MF) and biological process (BP) extracted from UniProt. For Arabidopsis proteins, they are also linked to their sources in The Arabidopsis Information Resource (TAIR) database (www.arabidopsis.org). The database will be curated and updated continuously to include additional members or new functions as experimental evidence surface in the future.

### Database design and interface

**Figure 3:**
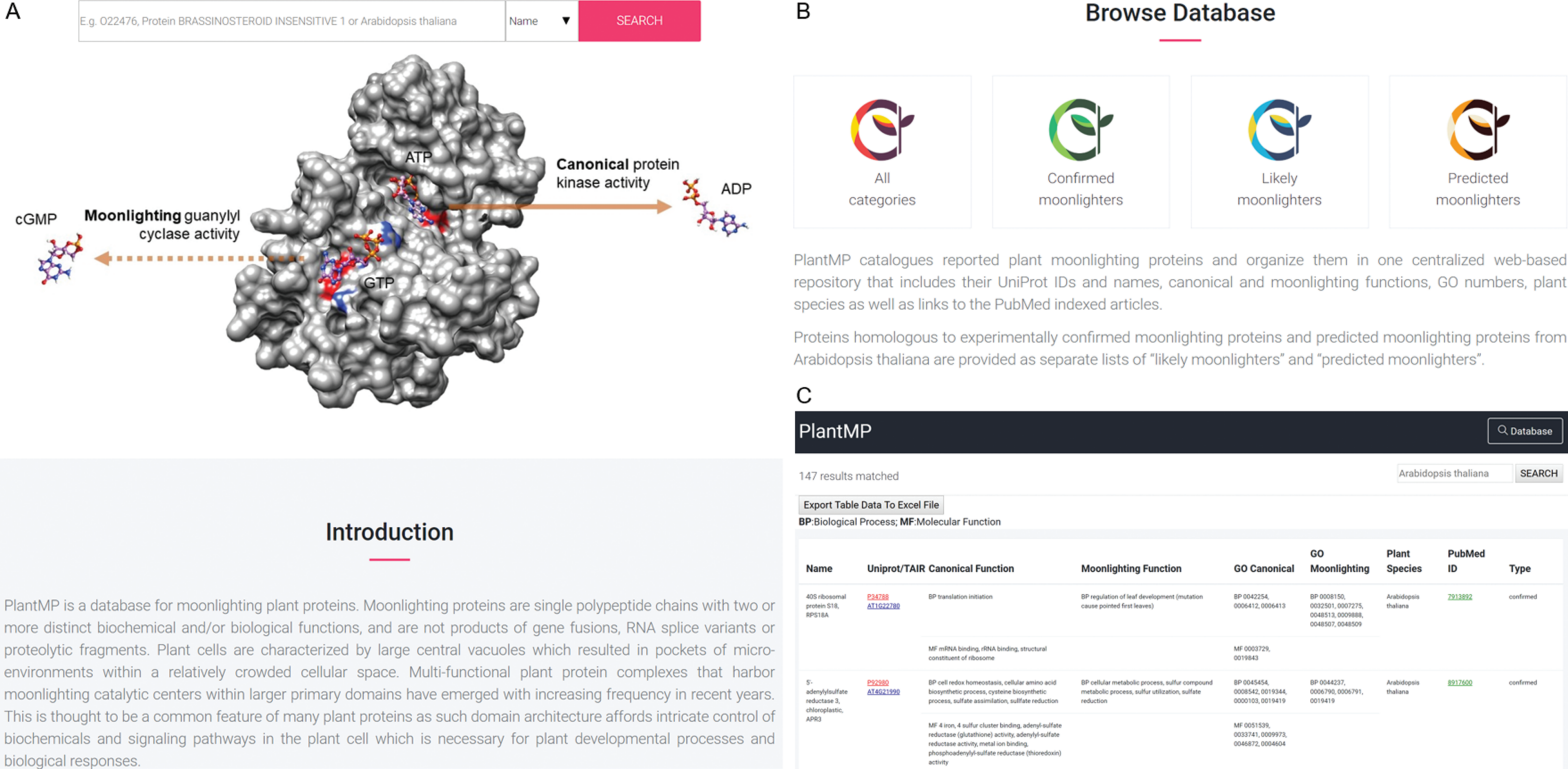
Layout of the PlantMP web server. Screenshot of the home page (A) beginning with a text search box that allows experienced users to quickly query and retrieve proteins of interest from three search options (protein name, UniProt ID and plant species) and then followed by a 3D model of a representative moonlighting protein (BRI1, UniProt ID: O22476) (14) docked with ATP and GTP and accompanied by clearly labeled primary and moonlighting catalytic activities to illustrate the function of the database. An introduction section that contains a brief description of moonlighting proteins and the database is provided to aid first time users navigate and extract information from the database. Following the Introduction is the `Browse Database’ section (B) where users can select from one of four lists of proteins: (1) All categories, (2) Confirmed moonlighters, (3) Likely moonlighters and (4) Predicted moonlighters. In the result page (C), moonlighting proteins in the PlantMP database contain UniProt IDs and names, canonical and moonlighting functions, GO numbers, plant species and links associated to the PubMed articles. Included in the canonical and moonlighting functions are their MF and BP extracted from UniProt (17). The website allows users to select desired proteins and export their results as Excel.

The website features a prominent top-aligned banner that houses the name of the database, a text search box, a 3D model of a representative moonlighting protein (BRI1, UniProt ID: O22476) (14) as well as `Introduction’ and `Browse Database’ section headers. The text search box allows experienced users to quickly query and retrieve proteins of interest from three search options: protein name, UniProt ID and plant species. The search box contains drop down suggestions to aid users identify proteins of interest. Following the search box is a 3D model of BRI1 docked with ATP and GTP and accompanied by clearly labeled primary and moonlighting catalytic activities to illustrate the function of the database. An introduction section that contains a brief description of moonlighting proteins and the database is provided to aid first time users navigate and extract information from the database. Following the introduction is the `Browse Database’ section where users can select from one of four lists of proteins: (i) All categories, (ii) Confirmed moonlighters, (iii) Likely moonlighters and (iv) Predicted moonlighters. The website allows users to select desired proteins and export their results as Excel. Currently, PlantMP contains 110 plant moonlighting proteins, 10 `likely moonlighters’ and 27 `predicted moonlighters’ (Figure 3).

## Results and discussion

Plant MP is available at https://www.plantmp.com without registration or license. While moonlighting proteins from human and other animal systems are well-characterized, those from plants are, however, ambiguous and under-represented in existing databases. PlantMP is to-date the most comprehensive catalog of plant moonlighting proteins featuring proteins from *A. thaliana*, crops and other non-model plants. Moonlighting proteins not found in existing databases such as those from *Pharbitis nil* and *Hippeastrum hybridum* whose catalytic activities are linked to phytochrome controlled photoperiodic flower induction and pathogen infection respectively (18, 19), are included here. Also featured are moonlighting proteins from crops such as tomato, carrot and rice. PlantMP is an initial effort to catalog plant moonlighters and will require a community-driven effort to make this database as comprehensive as possible. As such, we welcome users to reach us through the email provided at the bottom of the webpage to suggest plant proteins that warrant inclusion in our database, and our team will validate the request and update the database accordingly. Additionally, we are also exploring other repositories to identify plant moonlighters and conduct periodic updates on the database as new experimental evidence surface. A more focused plant-specific database allows less prominent moonlighting proteins or those from non-model plants to be highlighted especially since they may have potential for horticulture innovations and, consequently, added commercial value.

PlantMP is also inclusive as it accommodates ambiguous proteins or those whose functions/roles are unclear in separate categories of `likely’ and `predicted’ moonlighting proteins. Proteins in the `predicted’ list were identified using previously published search motifs: [KS][YF][GCS][VIL][VILFG][DVIL][VILADG][EPVIL][DVIL][TVIL][WST][PDRG][KEG][KR]x{2,3}[DHSE] for GC (13) and [R]X{5,20}[RKS][YFW][DE][VIL]X{4}[VIL]X{4}[KR]X{1,3}[DE] for AC (20). As one of the first search terms created for the discovery of plant GCs/ACs, these manually curated motifs were made stringent to reduce false positives, hence the inclusion of their identified candidates in our database. Other more relaxed versions of the search motifs have since been reported and applied (12, 21–23). Recently created prediction tools, GCPred (http://gcpred.com) (24) and ACPred (http://gcpred.com/acpred) (25), have considered more relaxed versions of the search motifs in their algorithms and used scores assigned to individual hits that are based on the physicochemical parameters of experimentally validated proteins instead, as measure of confidence. New hits obtained from predictions by currently available web servers are not included here because they have not been published nor validated experimentally or computationally. However, if users find new hits that are not experimentally proven but received good computational support that warrant their inclusion as `predicted moonlighters’, they can submit their requests to us for inclusion in our database. Given the increasing number of reported moonlighting plant proteins (12, 26, 27), an exclusive database is therefore necessary in order to reveal the features and nature of such proteins many of which are unique to the plant cell. Organizing plant moonlighting proteins in one platform enables researchers to conveniently harvest plant-specific data such as the MFs, biological roles and structural features essential for hypothesis formulation in basic research and for biotechnological innovations.

## Authors contributions

A.W. conceived and supervised this research. B.S. coordinated the project. B.S., Z.Q., T.L. and Y.Z. collected, compiled and analyzed the data and developed the web interface of the database. A.W. wrote the manuscript. All authors read and approved the final version of the manuscript.

## Notes

**Database URL:** https://www.plantmp.com
